# M2 macrophage-derived exosomal miR-26b-5p regulates macrophage polarization and chondrocyte hypertrophy by targeting TLR3 and COL10A1 to alleviate osteoarthritis

**DOI:** 10.1186/s12951-024-02336-4

**Published:** 2024-02-19

**Authors:** Yufan Qian, Genglei Chu, Lei Zhang, Zhikai Wu, Qiuyuan Wang, Jiong Jiong Guo, Feng Zhou

**Affiliations:** 1 Department of Orthopaedics, The First Affiliated Hospital of Soochow University, No. 899 Ping Hai Road, Suzhou, Jiangsu China; 2 Orthopedic Institute, Medical College, Soochow University, Suzhou, Jiangsu China

**Keywords:** Osteoarthritis, Exosomal miR-26b-5p, Macrophage polarization, Chondrocyte hypertrophy, Pain behavior

## Abstract

Osteoarthritis (OA) is one of the most prevalent chronic musculoskeletal diseases among the elderly population. In this study, macrophage-derived exosomes were isolated and identified. Exosomes were subjected to microRNA (miRNA) sequencing and bioinformatic analysis, and differentially expressed miRNAs were verified. miR-26b-5p target genes were confirmed through target-site mutation combined with a dual-luciferase reporter assay. The effects of miR-26b-5p on macrophage polarization and chondrocyte hypertrophy were assessed in vitro. miR-26b-5p agomir was applied to mice with OA induced by anterior cruciate ligament transection (ACLT). The therapeutic effects of miR-26b-5p were evaluated via pain behavior experiments and histological observations. In vitro, miR-26b-5p repolarized M1 macrophages to an anti-inflammatory M2 type by targeting the TLR3 signaling pathway. miR-26b-5p could target COL10A1, further inhibiting chondrocyte hypertrophy induced by M1 macrophage-conditioned medium (M1-CM). In vivo, miR-26b-5p agomir ameliorated gait abnormalities and mechanical allodynia in OA mice. miR-26b-5p treatment attenuated synovitis and cartilage degeneration, thereby delaying OA progression. In conclusion, M2 macrophage-derived exosomal miR-26b-5p could protect articular cartilage and ameliorate gait abnormalities in OA mice by targeting TLR3 and COL10A1. miR-26b-5p further affected macrophage polarization and chondrocyte hypertrophy. Thus, this exosomal miR-26b-5p-based strategy might be a potential method for OA treatment.

**Graphical Abstract:**

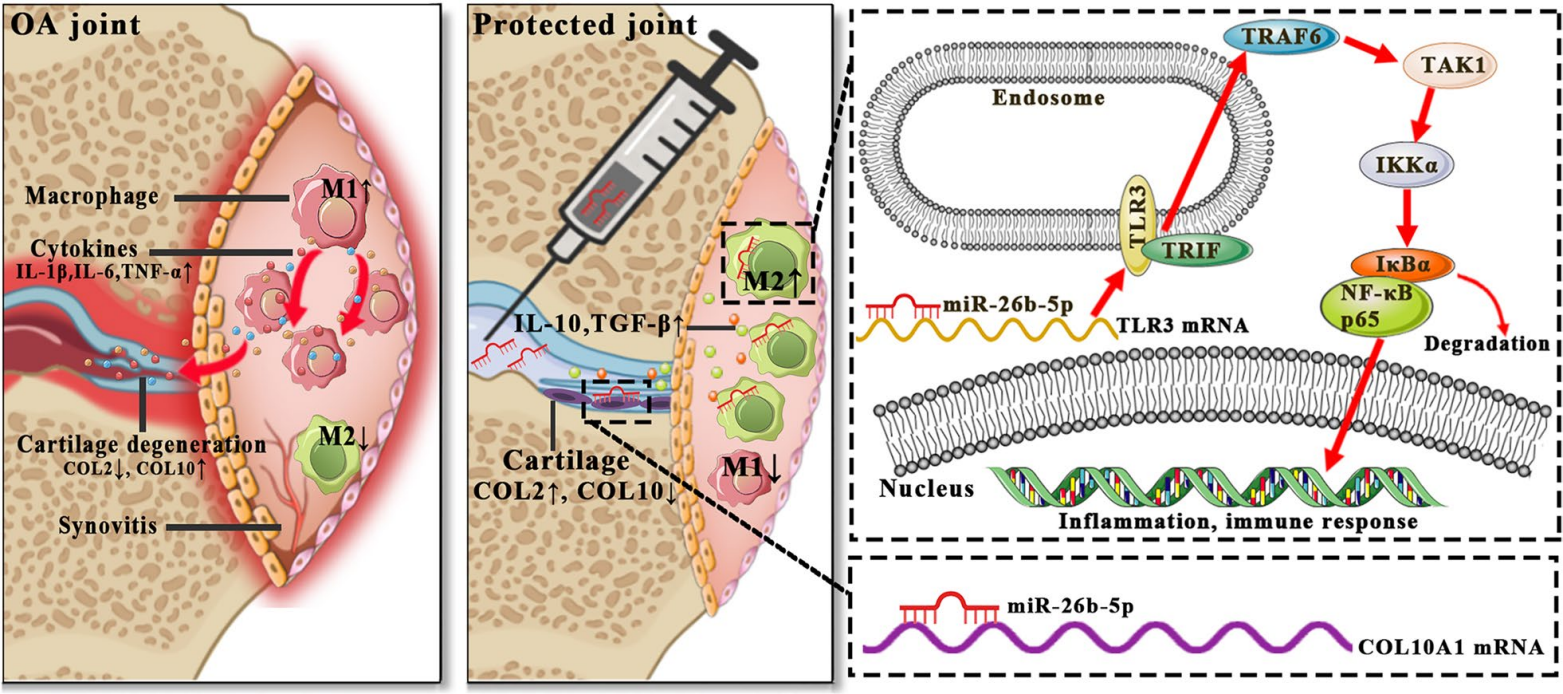

**Supplementary Information:**

The online version contains supplementary material available at 10.1186/s12951-024-02336-4.

## Introduction

Osteoarthritis (OA), the most prevalent joint disease among the elderly, affects more than 500 million individuals worldwide [1]. As a leading factor causing lower extremity disability, OA can cause joint pain and stiffness, which seriously affects the patient’s quality of life [2]. With the global population aging, the incidence of OA is rising, leading to increased economic burdens on families and society [3]. Therefore, there is a need to enhance our understanding of OA pathogenesis and develop new treatments.

Synovial macrophages play an important role in the pathogenesis and progression of OA [4]. They can be polarized into proinflammatory M1 or anti-inflammatory M2 phenotypes in response to different stimuli. M1 macrophages are activated through toll-like receptors (TLRs) by interferon-γ (IFN-γ) and lipopolysaccharide (LPS). M1 macrophages produce inducible nitric oxide synthase (iNOS), prostaglandin-endoperoxide synthase 2 (PTGS2), and proinflammatory cytokines such as IL-1β, IL-6, and TNF-α, leading to cartilage degeneration [5, 6]. IL-4, IL-10, and IL-13 can induce macrophage polarization into the M2 phenotype [7]. M2 macrophages secrete IL-10, arginase-1 (Arg-1), and transforming growth factor-β (TGF-β), which inhibit inflammation and promote tissue repair [8].

Macrophages in normal synovial tissues are usually quiescent, while in OA joints, they are predominantly polarized into proinflammatory M1 phenotype [9]. This activation of M1 macrophages contributes to a self-perpetuating cycle of synovial inflammation [10]. Hence, reprogramming synovial macrophages from M1 to the anti-inflammatory M2 phenotype holds promise for alleviating synovitis and attenuating cartilage degeneration, offering a potential therapeutic approach for managing OA.

Exosomes, small vesicles with a lipid bilayer structure, contain abundant microRNAs (miRNAs) [11, 12]. miRNAs regulate gene expression by influencing mRNA degradation or translation [13]. Certain miRNAs, such as miR-92a, miR-483-5p, miR-125b, and miR-320, have been implicated in the progression of OA, including chondrocyte proliferation, differentiation, hypertrophy, and cartilage matrix degradation [14, 15]. These findings suggest that miRNAs may have therapeutic potential for OA.

M2 macrophage-derived exosomes can suppress inflammation, thus helping to alleviate joint inflammation and promote cartilage repair [16]. miRNAs are abundantly found in M2 macrophage-derived exosomes. miR-124 derived from M2 macrophage exosomes has shown protective effects in brain ischemia–reperfusion injury [17]. However, the therapeutic potential of miRNAs from M2 macrophage exosomes in treating OA remains unclear. In this study, we sequenced miRNAs in macrophage exosomes to identify potential miRNAs for treating OA. Our findings will enhance understanding of the effectiveness of specific miRNAs from M2 macrophage exosomes in OA therapy.

## Results

### Isolation and identification of macrophage exosomes

**Fig. 1:**
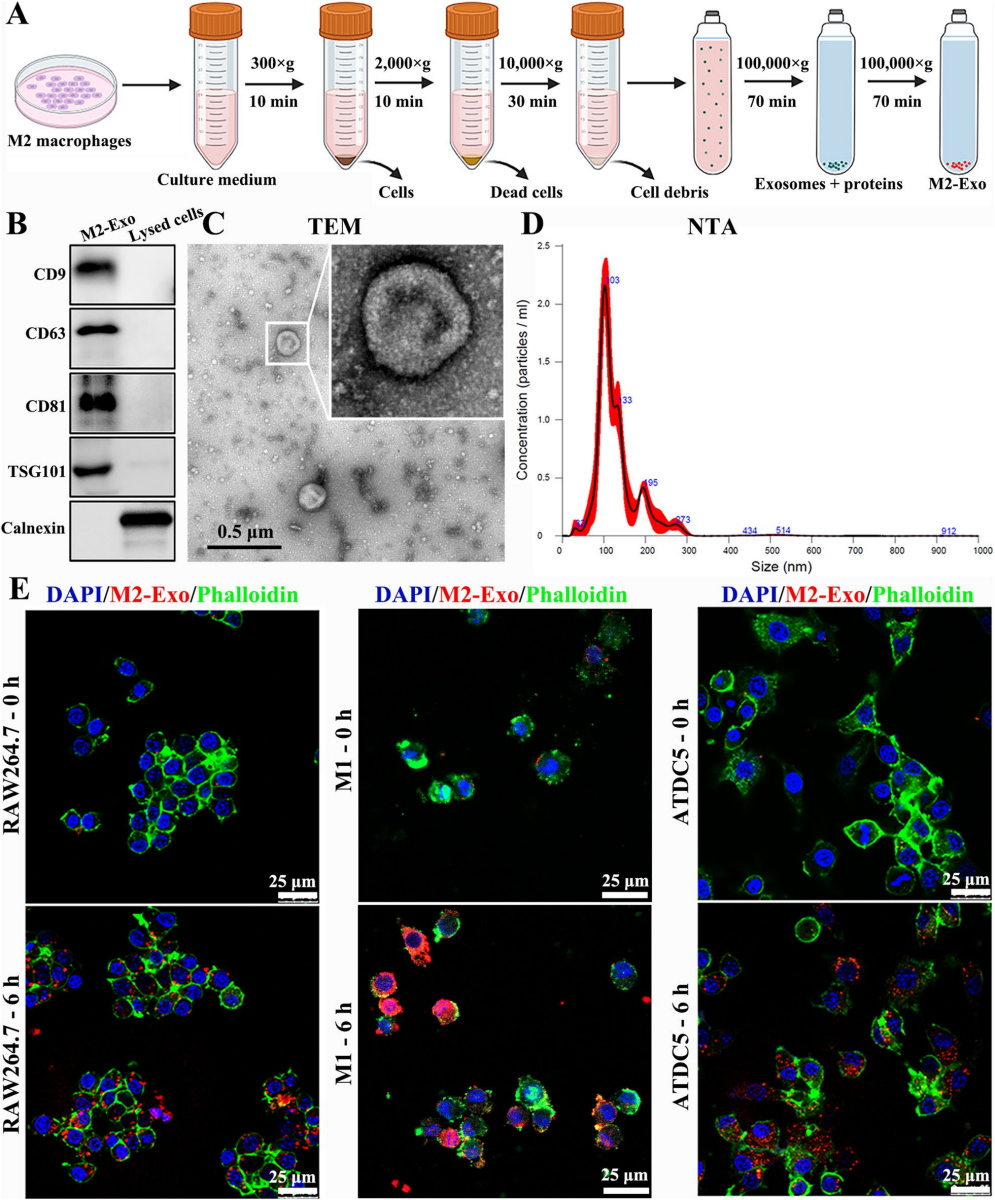
Identification and cellular uptake of M2 macrophage exosomes. **A** Flow chart of M2 macrophage exosome isolation. **B** Marker proteins of exosomes detected by western blotting. M2 macrophage-derived exosomes were characterized with TEM (**C**) and NTA (**D**). **E** Cellular uptake of PKH26-labeled exosomes in RAW264.7, M1 macrophages, and ATDC5 cells

The process of obtaining exosomes is shown in Fig. 1A. The results indicated that TSG101 and HSP70 were enriched in macrophage-derived exosomes compared with those in lysed cells (Fig. 1B). M2 macrophage-derived exosomes were further analyzed with transmission electron microscope (TEM) and nanoparticle tracking analysis (NTA). Typical cup-shaped vesicles were observed (Fig. 1C), and most of them had a size range of 30–200 nm (Fig. 1D). These data indicated that exosomes in the cell supernatant were successfully isolated.

### Exosome labeling and cellular uptake

M2 macrophage-derived exosomes were first labeled with PKH26, a red fluorescent lipophilic dye, to confirm whether macrophages and chondrocytes could uptake exosomes. RAW264.7 and ATDC5 cells were incubated with the exosomes. Red-fluorescent-labeled M2 exosomes were detected in the cytoplasm in RAW264.7, M1 macrophages, and ATDC5 cells (Fig. 1E), indicating that exosomes could be internalized.

### Exosomal miRNA expression profiles and analysis

**Fig. 2:**
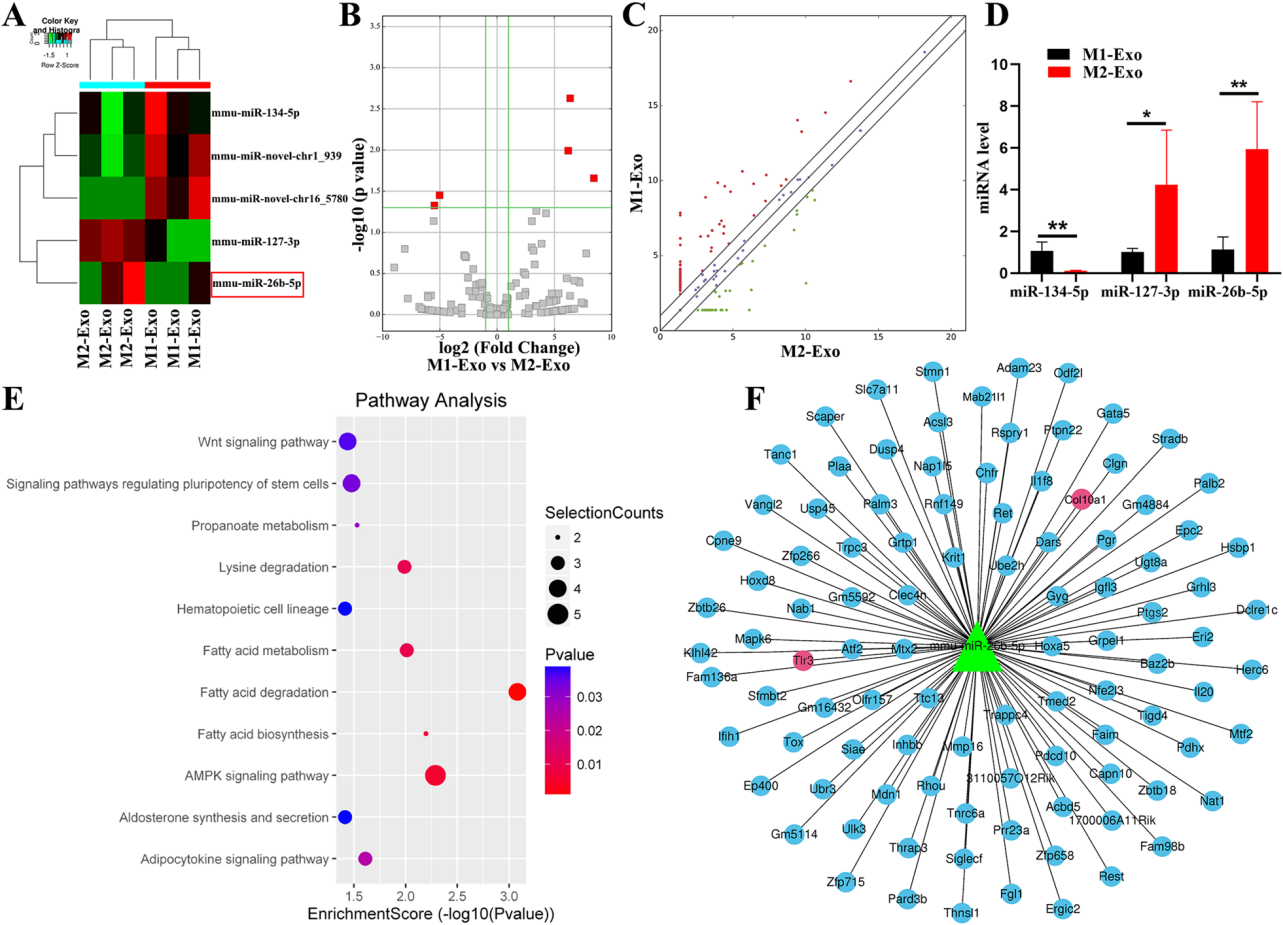
Exosomal miRNA expression profiles and bioinformatic analysis. **A** Hierarchical clustering showing a distinguishable miRNA between M1- and M2-derived exosomes. Volcano plot (**B**) and scatter plot (**C**) of differentially expressed exosomal miRNA. **D** Differentially expressed miRNA was verified via qRT-PCR, and mRNA fold-change was normalized to U6. **E** KEGG pathways of the differentially expressed miRNA. **F** Identified target genes of miR-26b-5p. *p < 0.05, **p < 0.01

An Illumina HiSeq4000 sequencer was used to analyze the profiles of miRNAs in M1/M2 macrophage-derived exosomes. The exosomal miRNA expression profiling is listed in Additional file 1: Figure S1, and the differentially expressed miRNAs in M1/M2 macrophage-derived exosomes are shown in Fig. 2A–C. Among these miRNAs, miR-127-3p and miR-26b-5p were significantly upregulated (fold-change > 2, p < 0.05), while miR-134-5p was downregulated (fold-change <  − 2, p < 0.05) in M2 macrophage-derived exosomes compared with those in M1 exosomes. Furthermore, qRT-PCR verified that these miRNAs were indeed significantly and differentially expressed (Fig. 2D). Meanwhile, the differentially expressed miRNAs were subjected to pathway analysis, which is a functional analysis of genes mapped to KEGG pathways (Fig. 2E).

Among the differentially expressed miRNAs, we focused our investigation on miR-26b-5p. Firstly, it has been reported that exosomal miR-26b-5p can regulate M1 macrophage polarization by inactivating the TLR pathway [18]. Additionally, miR-26b-5p has been shown to have a protective effect on chondrocytes and holds potential for the treatment of OA [19, 20]. In the present study, targets of miR-26b-5p were identified from multiple miRNA prediction databases, including TargetScan, PicTar, and miRanda. Among the predicted target genes of miR-26b-5p (Fig. 2F), TLR3 and COL10A1 were chosen for further investigation based on their significance. The TLR3 signaling pathway is known to be involved in M1 macrophage polarization [21], while COL10A1 is a recognized marker for hypertrophic chondrocytes [22]. These findings indicate that miR-26b-5p may play a role in modulating macrophage polarization and chondrocyte hypertrophy in OA joints.

### miR-26b-5p regulates the TLR3 signaling pathway in macrophages

**Fig. 3:**
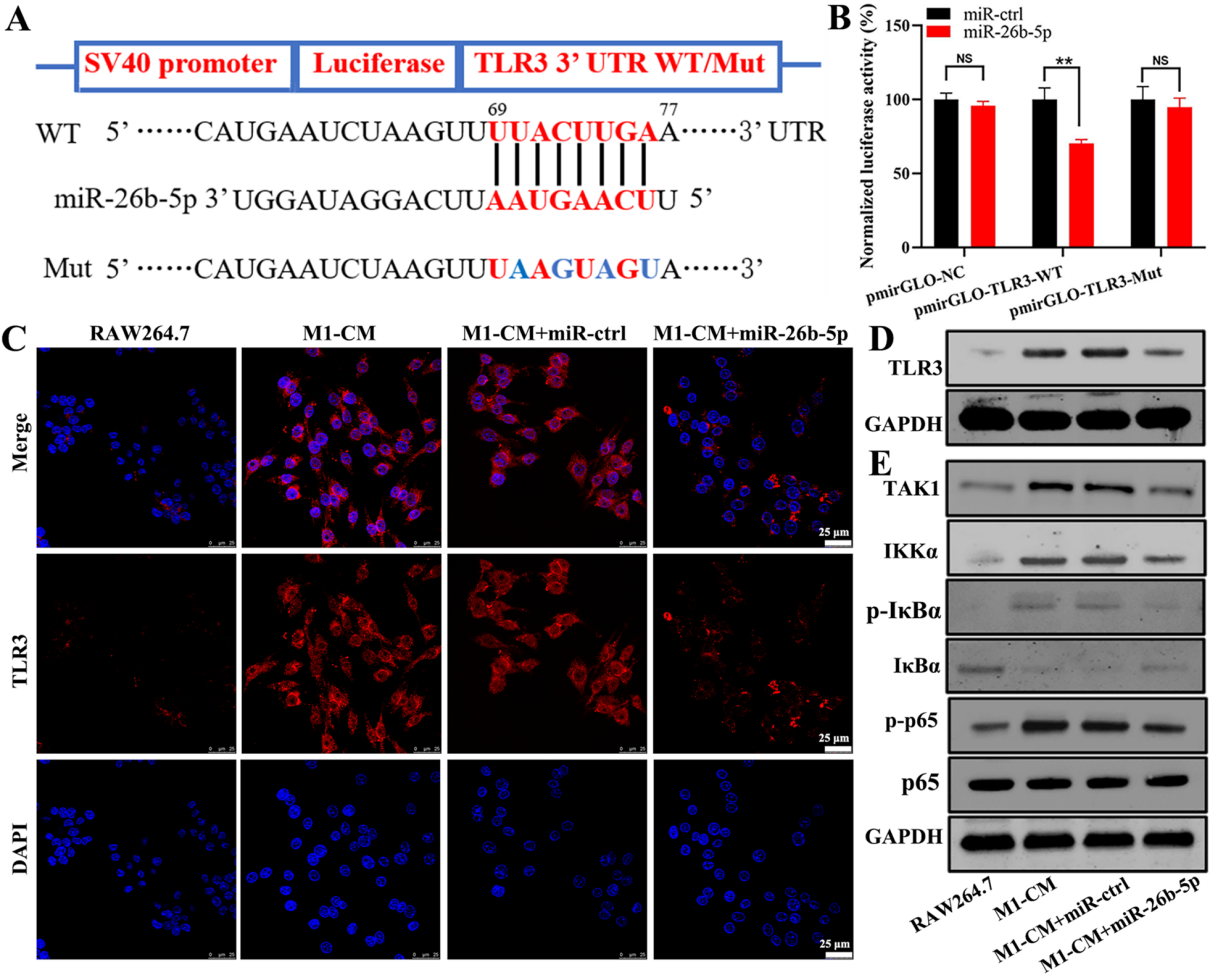
miR-26b-5p regulates TLR3 signaling pathway. **A** pmirGLO plasmids were constructed with WT or Mut 3’ UTR binding site sequences of TLR3. **B** Dual luciferase reporter assay showed the inhibition of miR-26b-5p on TLR3 expression in HEK293T cells. The expression of TLR3 after M1-CM stimulation was assessed by immunofluorescence staining (**C**) and western blotting (**D**). **E** Effects of miR-26b-5p overexpression on TLR3 signaling pathway. *p < 0.05, **p < 0.01

TLR3 is considered an endogenous sensor that can recognize double-stranded RNA (dsRNA) from viruses, degraded bacteria, damaged tissues and necrotic cells [23]. It is also an important pathway that promotes M1 macrophage polarization [21, 24]. The binding site of miR-26b-5p in the 3ʹ UTR of TLR3 is shown in Fig. 3A. The pmirGLO plasmids vectors mainly contained the SV40 promotor, luciferase reporter genes, and wild‐type (WT) or mutant (Mut) 3ʹ UTR binding site sequences of TLR3. The WT/Mut plasmids were co‐transfected with miR-26b-5p into the HEK293T cells. The results revealed that miR-26b-5p overexpression significantly suppressed the luciferase activity of the reporter gene in the pmirGLO-WT group. However, the inhibition was decreased because of the binding site mutations (Fig. 3B).

Multiple cytokine concentrations were detected using a Luminex liquid chip. Results showed that M1 macrophage-conditioned medium (M1-CM) had higher inflammatory cytokines, including IL-1β, IL-6, TNF-α, and IFN-γ (Additional file 1: Table S2). Then, the lentivirus-transfected RAW264.7 cell line is constructed (Additional file 1: Figure S2A, B). Immunofluorescence and western blotting results showed that M1-CM stimulation could promote the TLR3 expression, which was inhibited by the miR-26b-5p overexpression (Fig. 3C, D). The exogenous TLR3 ligand polyinosinic–polycytidylic acid (poly(I:C)) at a concentration of 10 μg/ml was used to activate the TLR3 signaling pathway [25]. Western blotting results demonstrated that the TLR3 signaling pathway was activated under poly(I:C) stimulation. This activation was specifically manifested in the increased expression of transforming growth factor β-activated kinase 1 (TAK1), IKKα, p-IκBα, and p-p65. Conversely, miR-26b-5p overexpression could inhibit these proteins compared with those in the miR-ctrl group (Fig. 3E). Therefore, miR-26b-5p could target TLR3, thereby suppressing the TLR3 signaling pathway.

### miR-26b-5p orchestrates macrophage polarization

**Fig. 4:**
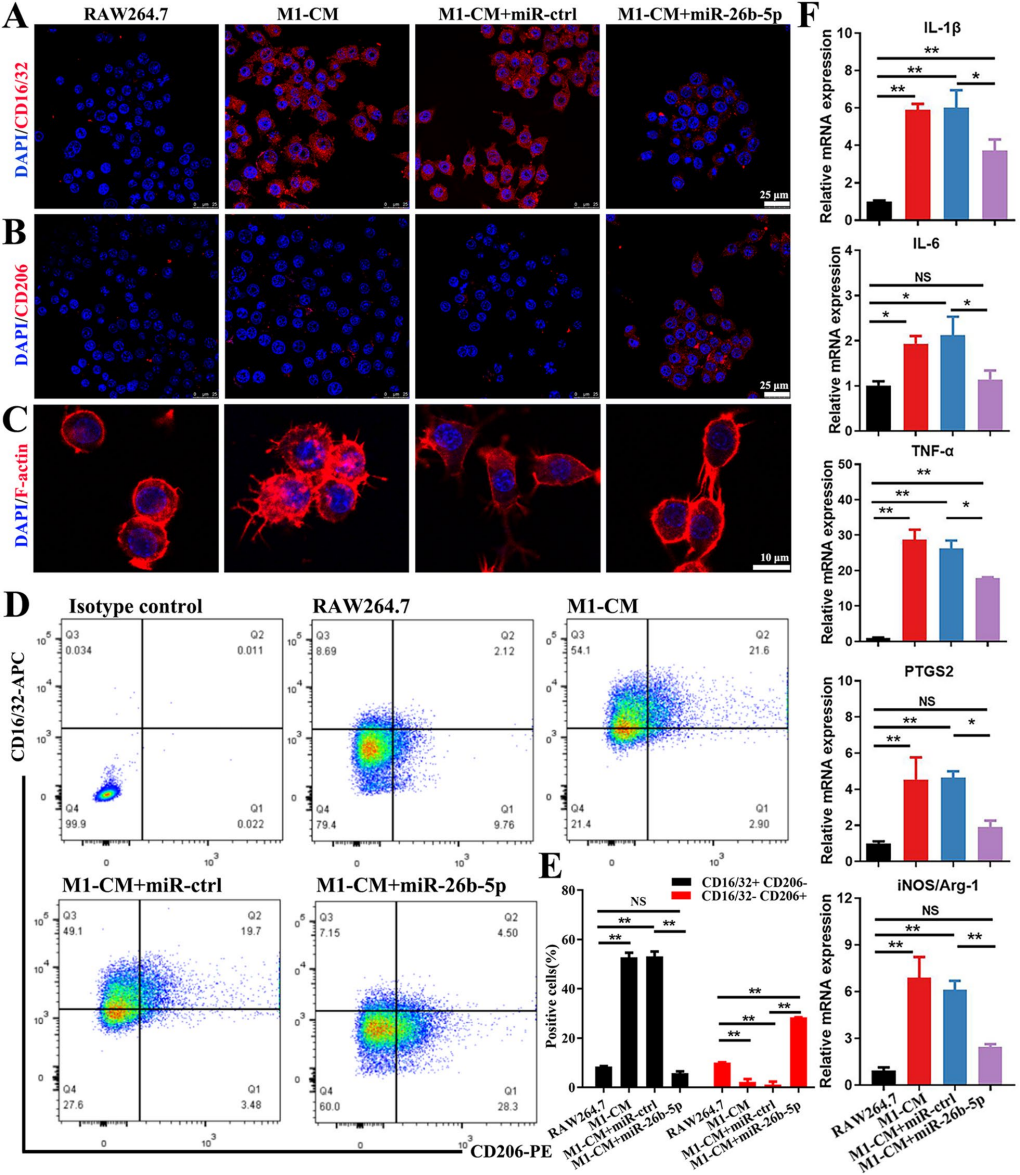
miR-26b-5p orchestrate macrophages polarization. M1/M2 macrophage marker CD16/32 (**A**) and CD206 (**B**) were assessed by immunostaining. **C** The effect of miR-26b-5p on the morphology of macrophages. **D** The percentage of M1 and M2 macrophages were assessed by flow cytometry. **E** Quantitative analysis of M1/M2 macrophages percentage. **F** Effects of miR-26b-5p om M1 macrophages related genes including IL-1β, IL-6, TNF-α, PTGS2, and iNOS/Arg-1 measured by qRT-PCR. *p < 0.05, **p < 0.01, NS means not significant

The effects of miR-26b-5p on macrophage repolarization were examined. Immunofluorescence results showed that miR-26b-5p overexpression suppressed CD16/32 expression and promoted CD206 expression (Fig. 4A, B). In Fig. 4C, macrophages treated with M1-CM showed flattened morphology with numerous pseudopodia. However, miR-26b-5p treatment reduced pseudopodia formation and induced a more elongated and spindle-like shape in the macrophages. These findings suggest that miR-26b-5p regulates the expression of surface molecules and morphology in macrophages, potentially influencing their polarization state. Flow cytometry analysis revealed that the percentage of M1 macrophages (CD206 negative and CD16/32 positive cells) in the M1-CM-stimulated group was 52.83 ± 3.19%. The M1 percentage was reduced to 5.78 ± 1.27% in miR-26b-5p overexpression group. Conversely, the percentage of M2 macrophages (CD206 positive and CD16/32 negative cells) was increased in the miR-26b-5p-treated group compared with that in the M1-CM-treated group (Fig. 4D, E). Moreover, miR-26b-5p reduced M1-related genes, including IL-1β, IL-6, TNF-α, PTGS2, and iNOS/Arg-1 (Fig. 4F). Therefore, miR-26b-5p could repolarize M1 macrophages to the M2 type, further reducing the secretion of harmful cytokines.

### miR-26b-5p inhibits M1-CM-induced chondrocyte hypertrophy

**Fig. 5:**
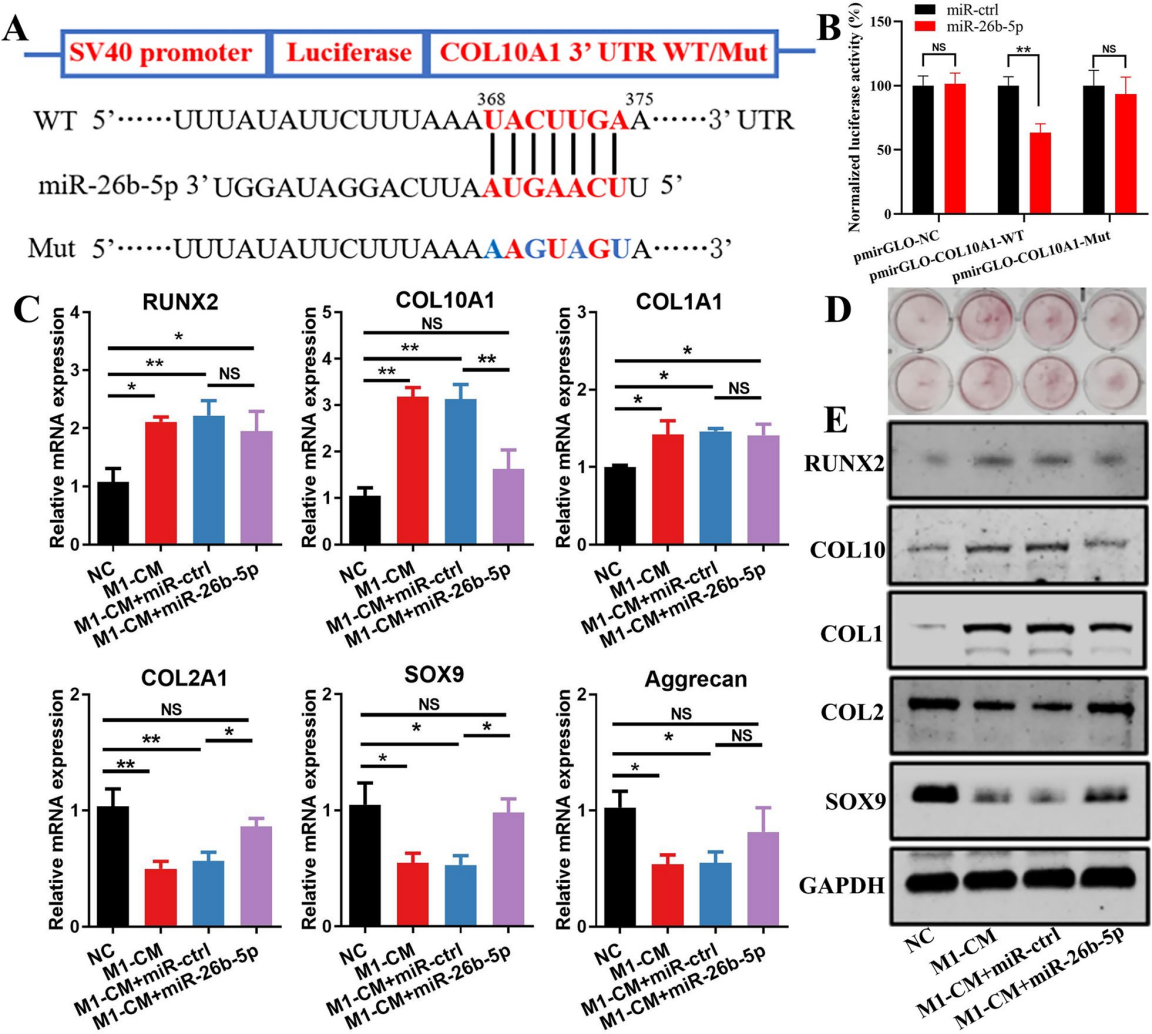
Inhibitory effect of miR-26b-5p on chondrocyte hypertrophy. **A** pmirGLO plasmids were constructed with WT or Mut 3′ UTR binding site sequences of COL10A1. **B** Inhibitory effect of miR-26b-5p on COL10A1 expression was assessed via a dual luciferase reporter assay. **C** Expression levels of RUNX2, COL10A1, COL1A1, COL2A1, SOX9, and Aggrecan in ADTC5 cells treated with M1-CM were examined via qRT-PCR. **D** ADTC5 cells were stimulated with M1-CM during chondrogenesis induction and stained with Alizarin red. **E** Western blotting analysis showed the protein expression of RUNX2, COL10, COL1, COL2, and SOX9. *p < 0.05, **p < 0.01, *NS* not significant

The pmirGLO plasmids containing the WT or Mut 3ʹ UTR binding site sequences of COL10A1 were constructed (Fig. 5A). The dual-luciferase reporter assay indicated that the miR-26b-5p overexpression significantly suppressed the luciferase activity in the pmirGLO-WT group but not in the pmirGLO-Mut group (Fig. 5B). In a previous study, M1-CM induces chondrocyte hypertrophy [6]. Hence, M1-CM was applied to ATDC5 cells during chondrogenesis induction in the present study. The lentivirus-transfected ATDC5 cell line is shown in Additional file 1: Figure S2C, D. The qRT-PCR results suggested that miR-26b-5p overexpression inhibited the gene expression of COL10A1 while promoted the gene expression of COL2A1 and SOX9 (Fig. 5C). Alizarin red staining revealed that miR-26b-5p inhibited the M1-CM-induced chondrocyte matrix mineralization (Fig. 5D). The results of western blotting analysis were consistent with those of qRT-PCR, indicating that miR-26b-5p suppressed the expression of COL10 and promoted the expression of COL2 and SOX9 proteins (Fig. 5E). Therefore, miR-26b-5p could suppress chondrocyte hypertrophy in vitro.

### Intra-articular injection of miR-26b-5p ameliorates gait abnormalities in anterior cruciate ligament transection (ACLT)-induced mice

**Fig. 6:**
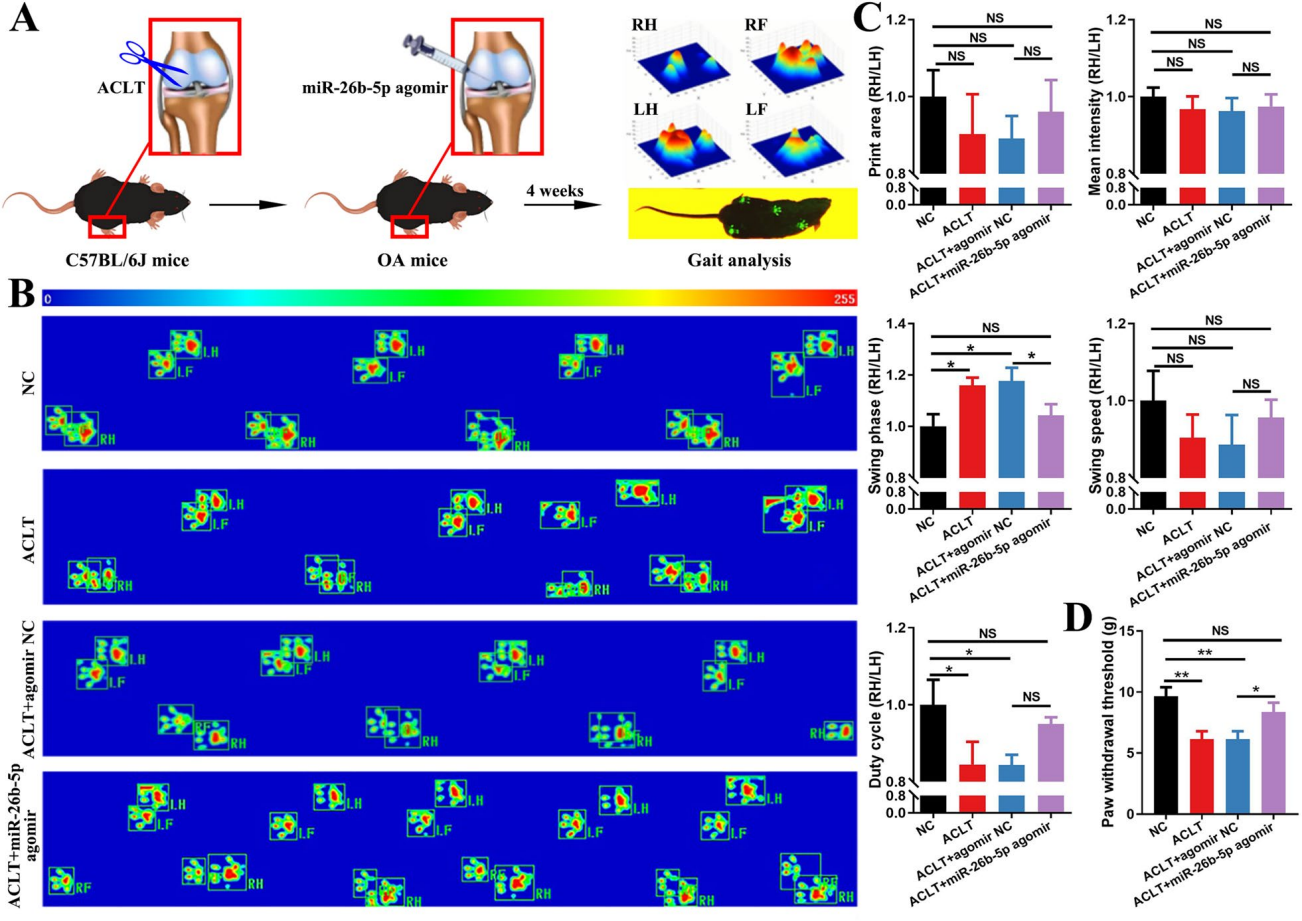
Effects of miR-26b-5p on pain behavior in OA mice. **A** Flow diagram showing the intra-articular injection of miR-26b-5p agomir and gait analysis. **B** Representative images of the footprints in various groups. **C** Quantitative analysis of gait parameters among different groups, presented as the ratio of RH/LH. **D** Evaluation of paw withdrawal threshold using a von Frey test. *p < 0.05, **p < 0.01, *NS* not significant

OA was induced in mice by using ACLT. Then, the specially labeled and chemically modified miR-26b-5p agomir was injected intra-articularly once a week for 4 weeks. CatWalk gait analysis was conducted to assess the foot gait of the mice at the end of injection (Fig. 6A). The representative images showing the gait and pressure distribution of the limbs in various groups are presented in Fig. 6B. Quantitative analysis revealed that the swing phase duration ratio of RH to LH was significantly reduced in the miR-26b-5p agomir-treated group compared with that in the agomir NC-treated group. miR-26b-5p agomir treatment could improve the print area, mean intensity, swing speed, and duty cycle to a limited extent (Fig. 6C). Furthermore, von Frey test demonstrated that miR-26b-5p agomir significantly reduced the paw withdrawal threshold in OA joints (Fig. 6D). Therefore, miR-26b-5p administration could partially improve gait patterns and tolerance to mechanical stimuli in OA mice.

### miR-26b-5p attenuates synovitis and chondrocyte hypertrophy to delay OA progression

**Fig. 7:**
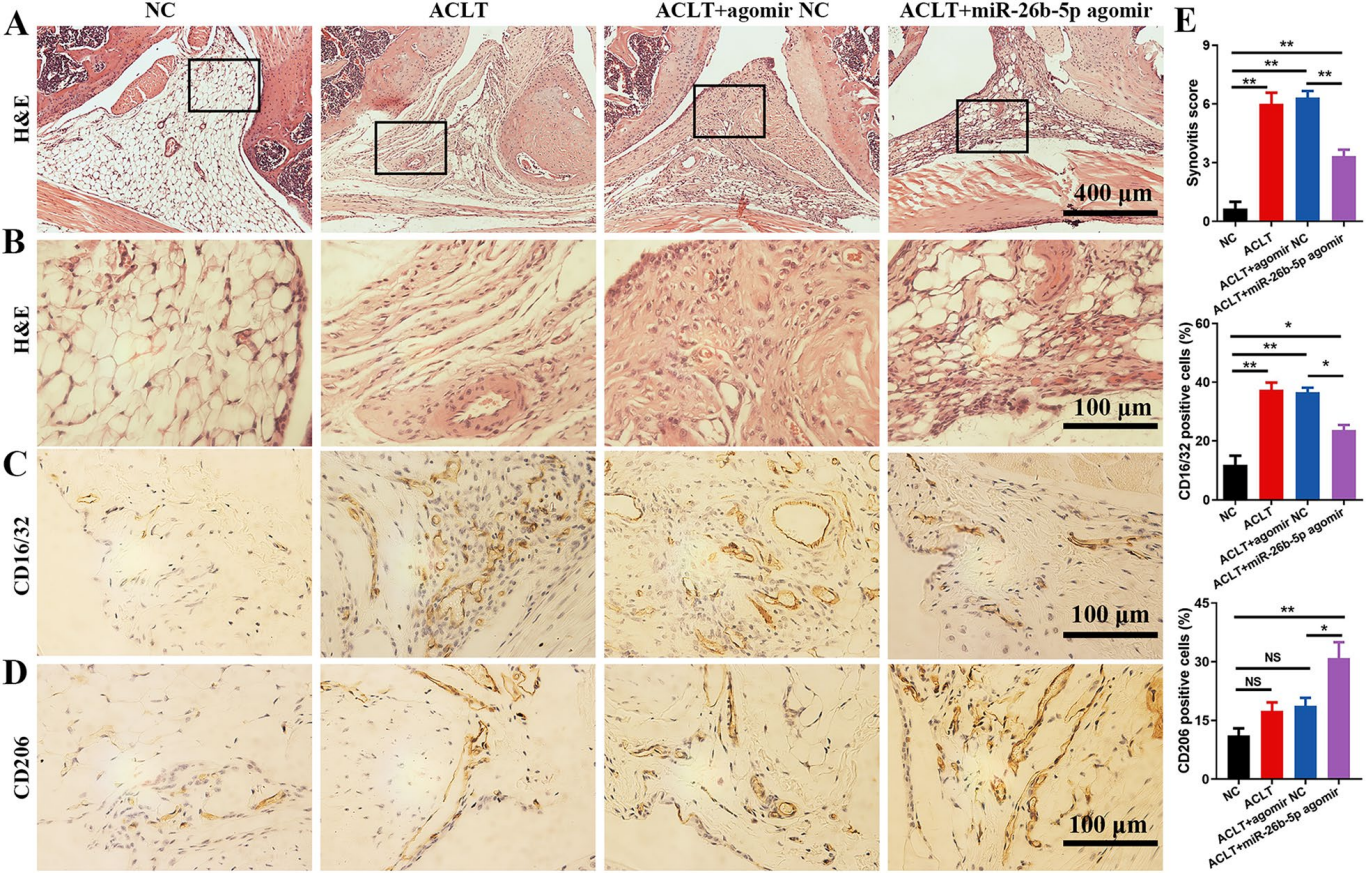
miR-26b-5p attenuated synovitis and regulated synovial macrophage polarization. H&E staining (**A**) and enlarged images (**B**) of mouse synovial tissues. **C** M1 macrophages (CD16/32-positive cells) in the synovial tissue. **D** M2 macrophages (CD206-positive cells) in the synovium. **E** Quantitative analysis of the synovitis score and the percentage of M1/M2-type macrophages. *p < 0.05, **p < 0.01, *NS* not significant

In ACLT-induced OA mice, synovitis was obviously observed in the increased synovial lining cells, angiogenesis, and inflammatory cell infiltration. Conversely, the intra-articular injection of miR-26b-5p agomir attenuated synovial inflammation compared with that of the OA mice (Fig. 7A, B). Immunohistochemistry was performed to examine the macrophage subtypes in the synovium. The results showed that the synovium of OA mice was mainly infiltrated by M1 macrophages accompanied by a small proportion of M2 macrophages. miR-26b-5p agomir treatment could decrease M1 macrophage infiltration and upregulate M2 macrophages in tge synovium (Fig. 7C, D). Quantitative analysis indicated that miR-26b-5p agomir significantly decreased the Krenn score, which was used to assess synovial inflammation. Moreover, miR-26b-5p efficiently decreased the proportion of M1 macrophages and improved the proportion of M2 macrophages in the synovium (Fig. 7E).

**Fig. 8:**
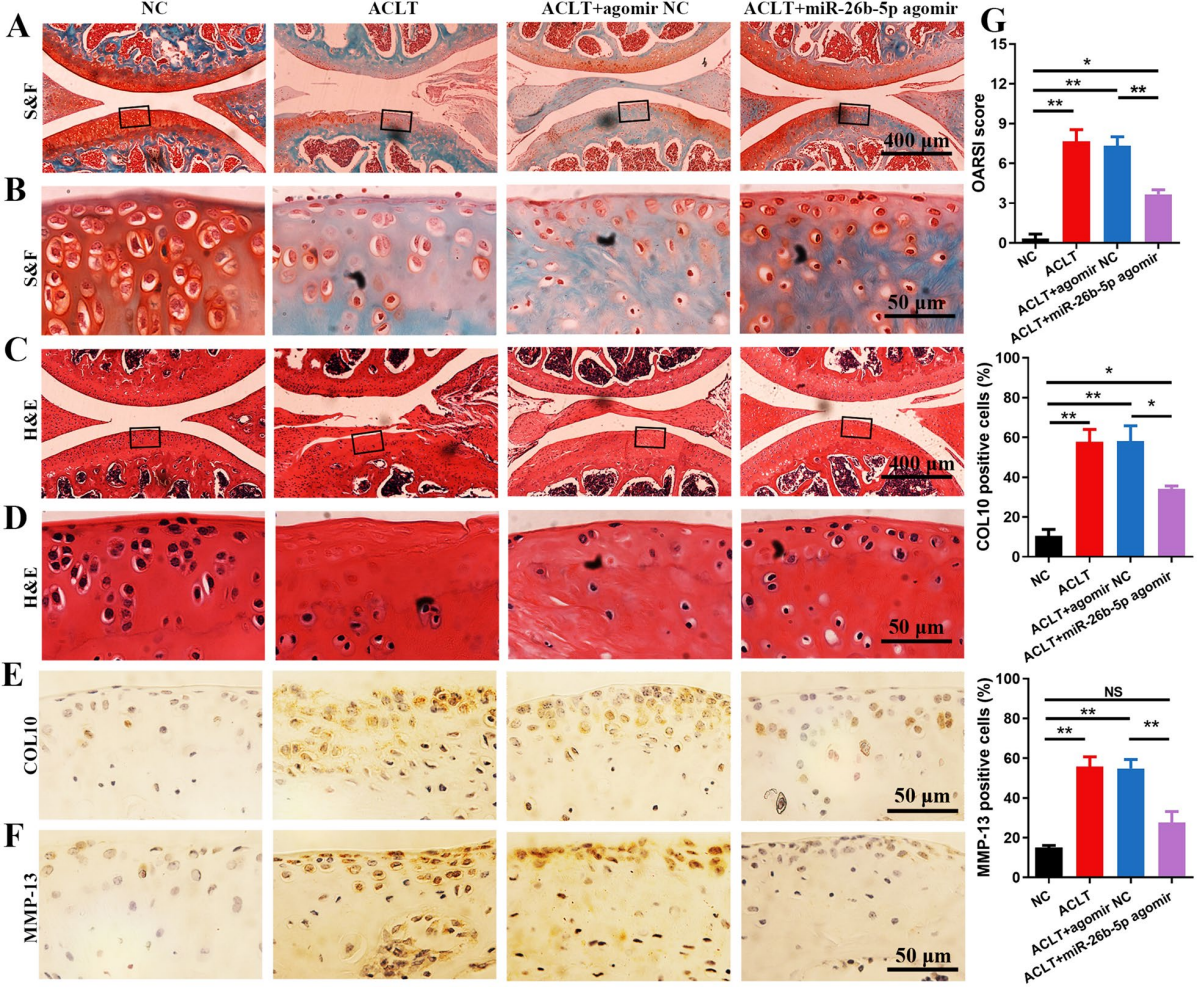
miR-26b-5p alleviated chondrocyte hypertrophy and delayed OA progression. S&F (**A**, **B**) and H&E (**C**, **D**) staining of cartilage in different groups of mice. COL10 (**E**) and MMP-13 (**F**) staining of the cartilage in various groups. **G** Quantitative analysis of OASRI score, COL10, and MMP-13 staining in cartilage. *p < 0.05, **p < 0.01, *NS* not significant

Safranin O-fast green (S&F) and hematoxylin and eosin (H&E) staining revealed the reduced cartilage matrix and thickness of the articular cartilage induced by ACLT surgery. Nevertheless, miR-26b-5p treatment could protect the articular cartilage in OA mice (Fig. 8A–D). Immunohistochemistry results indicated that miR-26b-5p intra-articular injection decreased the COL10 and MMP-13 expression in the articular cartilage (Fig. 8E, F). Quantitative analysis demonstrated that miR-26b-5p agomir significantly reduced the Osteoarthritis Research Society International (OARSI) score and inhibited the COL10 and MMP-13 expression compared with those in the other groups (Fig. 8G). Therefore, miR-26b-5p treatment could delay OA progression by regulating synovial macrophages and inhibiting chondrocyte hypertrophy.

## Discussion

In this study, M2 macrophage-derived exosomal miR-26b-5p was selected via high-throughput miRNA sequencing and bioinformatic analysis. The effects of miR-26b-5p on macrophage polarization and chondrocyte hypertrophy were assessed in vitro and in vivo. The results indicated that exosomal miR-26b-5p could repolarize proinflammatory M1 macrophages to an anti-inflammatory M2 type by targeting the TLR3 signaling pathway. Moreover, miR-26b-5p could inhibit articular cartilage hypertrophy by targeting COL10A1. Finally, miR-26b-5p ameliorated gait abnormalities and postponed OA progression. These data demonstrated that miR-26b-5p might be a potential OA treatment.

Therapeutic approaches, including nonpharmacologic and pharmacologic methods, have been applied to treat OA [26]. However, only a few strategies can efficiently delay OA progression because numerous factors are involved in slow cartilage degradation [27, 28]. As a component of the first line of immune defense, macrophages play vital roles during OA progression [29]. Previous study has shown that synovial macrophages could aggravate synovial inflammation and chondrocyte hypertrophy by secreting several proinflammatory cytokines such as IL-1β, IL-6 and TNF-α [30]. Thus, immunomodulatory strategies involving synovial macrophages may represent a potential treatment for OA. In this study, a synovial tissue was detected with mickle M1 macrophage infiltration accompanied by a small proportion of M2 macrophages. The results showed that miR-26b-5p could transform synovial proinflammatory M1 macrophages to an anti-inflammatory M2 type, thereby repairing the immune microenvironment in OA joints.

Exosomes play multiple roles in physiological and pathological processes by regulating intercellular communications. In OA progression, exosomes released from joint cells, including chondrocytes, osteoblasts, synovial fibroblasts, and macrophages, can be detected in the articular cavity [31]. The crosstalk between synovium-derived exosomes and joint cells in OA is yet to be fully understood, but a previous study reported that exosomes derived from IL-1β stimulated synovial fibroblasts (SFB) can dramatically induce articular cartilage degeneration [32]. On the contrary, exosomes produced by synovial mesenchymal stem cells (MSCs) can promote articular chondrocyte proliferation and migration; consequently, cartilage tissue regeneration is enhanced [33]. In addition to SFB and MSCs, other synovial cells, including macrophages, T cells, and endothelial cells, may participate in these processes; however, the function and mechanism of exosomes derived from these cells are largely unknown.

In addition to suppressing inflammation, M2 macrophage-derived exosomes have been shown to promote cartilage repair in arthritis by modulating macrophage re-polarization [16]. Moreover, M2 macrophage-derived exosomes have the potential to promote cartilage repair by increasing SOX and Aggrecan expression while reducing MMP13 levels [34]. M2 macrophage-derived exosomes carry bioactive molecules, such as miRNAs and proteins, that can modulate cellular activity and function. Particularly, the delivery of miRNAs by these exosomes has been found to play a crucial role in regulating chondrocyte proliferation, differentiation, and extracellular matrix synthesis, which are important for cartilage repair [35]. Our study focuses on investigating the specific miRNAs carried by M2 macrophage-derived exosomes and uncovering their underlying mechanisms in promoting cartilage repair.

miR-26b-5p is upregulated in ACL tissues of OA joints, indicating that it may participate in OA [36]. It also targets CH25H, thereby inactivating the TLR pathway and repressing M1 macrophage polarization. However, an ischemia/reperfusion (I/R) mouse model has been used, and the effects of miR-26b-5p on the TLR3 pathway have not been evaluated [18]. In another work, miR-26b-5p is significantly downregulated in OA cartilage and can regulate chondrocyte senescence by affecting asporin, demonstrating an miR-26b-5p-based therapeutic strategy for OA [19]. On the basis of sequencing and bioinformatic analysis, our work showed that miR-26b-5p was upregulated in M2 macrophage-derived exosomes and could target TLR3/COL10A1. The selection of TLR3 and COL10A1 was based on their known relevance to macrophage polarization and chondrocyte hypertrophy, respectively. However, it is important to consider the potential involvement of other target genes, and further studies are needed to explore their impact.

TLRs are pattern recognition receptors that sense multiple pathogen-associated molecular patterns (PAMPs), including microbial nucleic acids and surface glycoproteins. TLRs activate innate immunity. In the TLR family, TLR3 promotes intracellular signaling by recruiting the Toll/IL-1 receptor (TIR) domain-containing adaptor-inducing IFN-β (TRIF) adaptor to induce MyD88-independent signaling [37]. In response to dsRNA or polyI:C, TLR3 acts on several downstream signaling molecules including TNF receptor associated factor 6 (TRAF6). Furthermore, TRAF6 promotes the activation of TAK1 and IκB kinase (IKK) complex, causing the proteasomal degradation of IκBα. Phosphorylated p65 transfers into the nucleus and induces inflammation and immune regulation [38]. As a dsRNA receptor, TLR3 is traditionally generated during most viral infections, whereas degraded bacteria, damaged tissues, and necrotic cells can induce TLR3 expression [23]. TLR3 stimulation is important for M1 macrophage polarization [21]. Thus, TLR3 inhibition may be a potential strategy for skewing proinflammatory M1 macrophages to the anti-inflammatory M2 type. In this study, TLR3 was expressed under the stimulation of M1-CM, which contained various inflammatory factors. Under poly(I:C) treatment, the TAK1/IKKα/IκBα/NK-κB p65 axis was activated, promoting NF-κB p65 transcription. Therefore, miR-26b-5p overexpression could inhibit the TLR3 signaling pathway and consequently further repolarized M1 macrophages to the M2 type.

ATDC5 is a commonly used chondrogenic cell line that can be induced to chondrocytes via ITS stimulation [39]. During chondrogenesis, M1-CM is deployed to induce chondrocyte hypertrophy in ATDC5 cells. In our study, the conditioned media of macrophages were analyzed with a Luminex liquid chip for multicytokine detection. The concentration of inflammatory cytokines, including IL-1β, IL-6, TNF-a, and IFN-γ (Additional file 1: Table S2), in M1-CM was higher. On the one hand, these cytokines can cause articular cartilage hypertrophy; on the other hand, they can induce the surrounding quiescent macrophages to polarize to the M1 type [6, 9]. These two paths eventually accelerate OA progression. Conversely, the application of miR-26b-5p could simultaneously block the two pathogeneses by targeting TLR3 in macrophages and COL10A1 in chondrocytes.

M1 and M2 macrophages express different characteristic surface receptors. For example, M1 macrophages express high levels of MHC II molecules, CD16/32, CD80, and CD86 [40], and M2 macrophages highly express phagocytosis markers, including CD163 and CD206 [41]. In the present study, CD16/32 was chosen as a marker of M1 macrophages and CD206 of M2 macrophages as described previously [42]. Flow cytometry analysis revealed that some macrophages were positively stained with CD16/32 and CD206. These non-M1- and non-M2-phenotype cells may be in a wandering state between M1 and M2 macrophages [43]. Although the role of this macrophage subset remains unclear, this result indicated that M1/M2 macrophages could not be absolutely distinguished, and polarization could be a dynamic process.

We applied intra-articular injection, a commonly used method for OA treatment, to administer miRNA to the joints. Intra-articular injection can increase local drug concentration and bioavailability, minimize systemic exposure and adverse events, and reduce cost compared with those of systemic drug delivery [44]. In the present study, all mice did not suffer from joint infections, indicating the safety of intra-articular injection to a certain degree. The dosage and frequency of miRNA used in this work were based on previous studies [45, 46]. We applied miRNA agomir, which has been specially labeled and chemically modified, to regulate the biological functions of target genes by mimicking endogenous miRNA. We administered miRNA to target synovial macrophages and articular chondrocytes. However, we have yet to clarify whether miRNA could infiltrate the subchondral bone and affect bone remodeling or be absorbed into blood circulation.

In addition to ACLT, the destabilization of the medial meniscus (DMM) is a commonly used model of OA. However, a DMM-induced OA model shows lower levels of synovitis than ACLT does [39]. In the present study, an ACLT-induced OA model was applied to evaluate the effects of miR-26b-5p on synovitis more clearly. Our data showed that synovial inflammation was obvious in OA mice. In particular, the thickness of synovial lining cells and the number of blood vessels increased; furthermore, inflammatory cell infiltration in the synovium was enhanced. Hence, the OA mice had a significantly high Krenn score, which was designed to evaluate synovial inflammation. Under miR-26b-5p treatment, the M1/M2 macrophage phenotype shifted, and Krenn score obviously decreased.

Gait analysis and von Frey test are important behavioral analyses for measuring pain in patients with OA. In this study, the VisuGait system was deployed for gait analysis in accordance with previously described methods [47]. Several mouse gait-related parameters, including print area, pressure intensity of limb, swing phase duration, swing speed, and duty cycle, were obtained. Previous results of gait analysis in OA mice remain inconsistent, possibly because of variations in the operation method, analysis time point, kind and number of animals, and other aspects [47–50]. In this study, OA mice presented gait abnormalities induced by hyperalgesia in ACLT-induced joints. The results of gait analysis in this study were presented as the ipsilateral knee/contralateral knee to eliminate individual differences. Thus, miR-26b-5p treatment could ameliorate gait abnormalities to a certain degree while significantly reducing mechanical allodynia in OA mice.

## Conclusion

In conclusion, M2 macrophage-derived exosomal miR-26b-5p might be an effective treatment for OA. Intra-articular miR-26b-5p injection could alleviate synovial inflammation and cartilage degeneration, and the underlying mechanism likely involved miR-26b-5p-mediated macrophage repolarization and inhibition of chondrocyte hypertrophy by targeting TLR3/COL10A1. Thus, our study might serve as a basis for developing a potential strategy for OA treatment.

## Materials and methods

### Cells, media, and reagents

RAW264.7, a macrophage cell line, was cultured in Dulbecco’s minimum essential medium (DMEM; HyClone, Logan, USA) added with 10% fetal bovine serum (FBS; Gibco, New York, USA), 100 U/ml penicillin (Gibco, New York, USA), and 100 μg/ml streptomycin (Gibco, New York, USA). ATDC5, a chondrogenic cell line, was cultured in DMEM: nutrient mixture F12 (DMEM: F12; HyClone, Logan, USA) supplemented with 5% FBS. The cells were placed at 37 °C under humidified conditions with 5% CO_2_. The bacterial LPS of Escherichia coli and the recombinant mouse IFN-γ and IL-4 were purchased from PeproTech (Rocky Hill, USA). Exosome-depleted FBS was bought from System Biosciences (California, USA).

### Induction of macrophage polarization

RAW264.7 cells were seeded on culture plates, incubated at 37 °C overnight to adhere to the bottom of the plates. Cells were stimulated with a complete medium containing 100 ng/ml LPS plus 20 ng/ml IFN-γ 24 h to induce M1 polarization and 20 ng/ml IL-4 24 h to stimulate M2 polarization [6].

### Exosome isolation and identification

RAW264.7 cells were induced to an M1/M2 type for 24 h and cultured in an exosome-depleted FBS-containing complete medium. The supernatant was collected, and exosomes were obtained through ultracentrifugation [35]. Briefly, the supernatant was subjected to a series of differential centrifugation steps (300×*g* for 10 min, 2000×*g* for 10 min, and 10,000×*g* for 30 min) to remove intact cells and cell debris. Subsequently, the supernatant was centrifuged at 100,000×*g* at 4 °C for 70 min to isolate the proteins-containing exosomes. The exosomes were then purified by washing them with PBS and subjected to an additional centrifugation step at 100,000×*g* for 70 min.

The protein content of the exosomes was measured with a bicinchoninic acid (BCA) protein assay kit (Beyotime Biotechnology, Shanghai, China) referring to the manufacturer’s instructions. CD9, CD63, CD81, TSG101 and Calnexin (Proteintech, Chicago, USA), which were exosomal marker proteins, were assessed by western blotting as previously described [51]. M2 macrophage-derived exosomes were scanned using a TEM (JEM1400, Tokyo, Japan). The size and concentration of exosomes were analyzed through NTA (Nanosight NS300, Malvern, UK).

### Exosome labeling and cellular uptake

M2 macrophage-derived exosomes were labeled with fluorescent dyes referring to a previous study [52]. They were labeled using a PKH26 red fluorescent cell linker kit (Sigma-Aldrich, St. Louis, USA) in accordance with the manufacturer’s instructions. The labeled exosomes were washed and centrifuged at 100,000×*g* for 60 min. Then, the resuspended exosomes were deployed to RAW264.7 and ATDC5 cells for 6 h. After the supernatant was removed, FITC-conjugated phalloidin (Sigma-Aldrich, USA, St. Louis, USA) and 4,6-diamidino-2-phenylindole (DAPI; Beyotime Biotechnology, Shanghai, China) were used to stain the cytoskeleton and nuclei, respectively. The cells were washed with PBS twice and photographed with a confocal microscope (Leica TCS-SP5, Germany).

### Exosomal miRNA sequencing and analysis

High-throughput miRNA sequencing and subsequent bioinformatic analysis were performed by CloudSeq Biotech Co., Ltd. (Shanghai, China). The total RNA of exosomes was prepared and quantified using a NanoDrop ND-100 (Thermo Fisher Scientific, USA). Then, total RNA of each sample was used to prepare the miRNA sequencing library. The libraries were denatured as single-stranded DNA molecules, captured on Illumina flow cells, amplified in situ as clusters, and sequenced for 50 cycles on an Illumina HiSeq4000 sequencer (Illumina, CA, USA). Raw data were generated through sequencing, image analysis, base calling, and quality filtering by using an Illumina sequencer.

### RNA extraction, reverse transcription-PCR and real-time qPCR

The total RNA of cells was extracted using TRIzol reagent (Invitrogen, CA, USA), and NanoDrop ND-100 was used to measure the quality and concentrations of RNA. cDNA was synthesized with TaqMan reverse transcription reagents (Applied Biosystems, CA, USA). Real-time qPCR was performed using an ABI 7500 system (Applied Biosystems, CA, USA) in accordance with previously described methods [53]. The reverse transcription primers of miRNAs were prepared using the stem-loop method. The transcript levels of mRNA and miRNA were normalized to β-actin and U6, respectively. Gene expression levels were analyzed using 2^−ΔΔCt^ method. The primers used for these analyses are listed in Additional file 1: Table S1.

### Lentivirus preparation and construction of stably transfected cell lines

pHBLV-zsgreen-puro (miR-ctrl) and pHBLV-miR-26b-5p-zsgreen-puro (miR-26b-5p overexpression) plasmids were prepared by Hanbio Biotechnology Co., Ltd. (Shanghai, China). The prepared plasmids were transfected into HEK293T cells with LipoFiter^™^ 3.0 (liposomal transfection reagent). The virus supernatant was collected and filtered using a 0.45 μm cell strainer 48 h after transfection. The supernatant was further centrifuged to 72,000 g at 4 °C for 120 min and resuspended with fresh media for the following experiments. The titer of the concentrated lentivirus was measured reaching 2 × 10^8^ TU/ml.

RAW264.7 and ATDC5 cells were seeded in six-well plates (5 × 10^5^/well) and incubated overnight. Then, 6 μg/ml polybrene (Sigma-Aldrich, St. Louis, USA) was applied before transfection. The lentivirus-containing medium was changed with complete media 24 h after transfection, and fluorescence signal was observed using a fluorescent microscope 48 h after transfection. Green fluorescence indicated successful transfection in cells. Then, 4 μg/ml puromycin was added to the culture medium to kill untransfected cells. After about 7 days, the successfully transfected cells survived, and they were used for the following analysis.

### Dual-luciferase construction and reporter assay

pmirGLO dual-luciferase miRNA target expression vectors were prepared by GenePharma (Suzhou, China) to assess the miRNA activity. The 3ʹ UTR sequences of TLR3 and COL10A1 (wild type or mutant) were cloned in the vectors. The firefly luciferase reporter gene was controlled by an SV40 promoter, and Renilla luciferase was used as a control reporter for normalization. HEK293T cells with overexpressed miR-ctrl or miR-26b-5p were co‐transfected with various pmirGLO vectors for 24 h. Then, the luciferase activity in the lysates was assessed with a dual-luciferase reporter gene assay kit (Beyotime Biotechnology, Shanghai, China) in accordance with the manufacturer’s instruction.

### Cell co-culture experiments

RAW264.7 cells were polarized to an M1 type by stimulating with LPS and IFN-γ for 24 h. M1 macrophage supernatants were centrifugated at 1000×*g* for 5 min and diluted with a serum-free medium (1:1) to prepare M1-CM for the following experiments. The obtained M1-CM was deployed to RAW264.7 to evaluate the effects on macrophage polarization and added with insulin, transferrin, and selenous acid (ITS; Sigma-Aldrich, St. Louis, USA) to evaluate the chondrogenesis of ATDC5 cells [39]. M1-CM was analyzed with a Luminex liquid chip for multi-cytokine detection to assess the effects of miR-26b-5p on macrophage repolarization and chondrocyte hypertrophy.

### Immunofluorescence staining

RAW264.7 cells with or without miR-26b-5p overexpression were stimulated with M1-CM for 24 h. They were fixed in 4% paraformaldehyde containing 0.1% Triton X-100 (Sigma-Aldrich, St. Louis, USA), and 1% bovine serum albumin (BSA) was applied to block nonspecific binding. Afterward, the cells were incubated with antibodies, including CD16/32 (BD, CA, USA), CD206 (BD, CA, USA), and TLR3 (Abcam, Cambridge, MA, USA), overnight. They were further incubated with fluorescent secondary antibodies (Abcam, Alexa Fluor 594, Cambridge, MA, USA) and DAPI were transferred to cells and observed using a confocal fluorescence microscope (Leica TCS-SP5, Germany).

### Flow cytometry analysis of macrophage subsets

CD16/32 and CD 206 were chosen to respectively mark M1 and M2 phenotypes and to distinguish M1 and M2 macrophage populations. After being washed thrice with PBS, the polarized macrophages were digested and resuspended with PBS. Then, 5 μl of Alexa anti-CD206 (Fluor 647-conjugated, BD, CA, USA) and 5 μl of anti-CD16/32 (PE-conjugated, BD, CA, USA) were applied to evaluate the macrophage subsets. Alexa Fluor 647 isotype control (BD, CA, USA) and PE isotype control (BD, CA, USA) were transferred to exclude the cells with nonspecific staining. M1 macrophages were regarded as CD16/32-positive and CD206-negative cells, while M2 macrophages were denoted as CD206-positive and CD16/32-negative cells.

### Western blotting analysis

The cells were washed with PBS and lysed with a radioimmunoprecipitation assay buffer (RIPA; Millipore, MA, USA) containing protease and phosphatase inhibitors (Sigma-Aldrich, St. Louis, USA). The protein concentrations of cells were evaluated using a BCA protein assay kit. Then, 20 μg of proteins was separated through 10% SDS-PAGE and transferred to polyvinylidene fluoride (PVDF) membranes. The membranes were blocked with 5% nonfat milk for 2 h and incubated with primary antibodies overnight. The membranes were further incubated with fluorescent secondary antibodies and washed thrice with Tris-buffered saline with Tween (TBST). Fluorescent signals were photographed with LI-COR imaging systems (Lincoln, USA).

### Alizarin red staining

The chondrogenesis of ATDC5 cells was induced for 14 days. Then, the cells were fixed with 4% paraformaldehyde, incubated with 1% Alizarin red solution (Sigma-Aldrich, St. Louis, USA) for 30 min, [39] and washed with PBS twice to remove excess Alizarin red dye. Afterward, they were photographed under an optical microscope.

### Establishment of ACLT-induced OA mouse model

Animal experiments were approved by the ethics committee of the First Affiliated Hospital of Soochow University. Twenty-four 3-month-old male C57BL/6J mice obtained from Laboratory Animal Center of Soochow University were exposed under specific pathogen-free (SPF) conditions and fed with commercial food and water. OA was induced by applying ACLT, which can cause abnormal mechanical loading, to the right knee in accordance with previously described methods [54, 55]. An anterior drawer test was conducted to confirm complete transection, and a sham operation was made by opening the joint capsule and suturing the incision.

The mice were randomly and averagely split into four groups and assigned to four cages. The mice in group 1 were assigned in the control group and treated with sham operation. The mice in groups 2–4 were treated with ACLT to the right knee joints. The mice in group 2, 3, and 4 were also intra-articularly injected with PBS, 5 nmol agomir NC, and 5 nmol miR-26b-5p agomir, respectively. Intra-articular injection was administered once a week for 4 weeks [45, 46].

### Gait analysis

Gait analysis was performed using a gait analysis system as described previously [47]. In brief, the gait analysis system contains a fluorescent light tube along a glass plate runway. The mice were placed individually on the runway and allowed to walk across the runway freely. The gait of each mouse was recorded using a high-speed color camera and analyzed with the VisuGait software (Xinruan Co., Ltd., Shanghai, China). The swing phase duration (s) indicated the period when the paw was not touching the ground in a complete step cycle. The swing speed (m/s) was calculated by dividing the stride length by the duration of the swing phase. The duty cycle (%) was defined as the ratio between stance duration and complete step cycle duration [56]. The ratios of the affected right hind limb to the contralateral left hind limb (RH/LH) were calculated to eliminate individual differences [50].

### Von Frey withdrawal threshold testing

Von Frey filaments were applied to assess the secondary mechanical allodynia by measuring the withdrawal threshold in accordance with previously described methods [56]. The mice were placed in a transparent plexiglass chamber with a metal mesh floor for at least 10 min to acclimatize them before the test. The filaments were applied when the mouse stood still on all four paws and operated thrice with an inter-trial interval of 10 s. The response was considered positive when the mice flinched their paw more than once. Then, the paw withdrawal threshold (PWT) of the mouse was recorded.

### Histological observation

The mice were killed 4 weeks after the injection. The right knee joints of the mice in various groups were dissected and fixed with 4% paraformaldehyde for 48 h. The samples were decalcified in 10% EDTA for 3 weeks and embedded in paraffin. The sagittal sections of the medial compartment of the knee joints were cut at 4 μm for the following microstructural observations. They were further stained with S&F and H&E. The OARSI score was applied in accordance with previously described methods [57]. Synovitis was assessed using the Krenn scoring system [58]. Immunohistochemical staining was applied in accordance with a previous report [59].  Specifically, primary antibodies, including CD16/32, CD206, COL10, and MMP-13 (Abcam, Cambridge, MA, USA, dilution 1:200) were applied and incubated at 4 °C overnight. A secondary antibody was incubated at 25 °C for 1 h. The samples were stained with diaminobenzene (Dako, North Sydney, NSW, Australia) and hematoxylin (Sigma-Aldrich, St. Louis, USA). The immunohistochemically stained samples were then photographed under a microscope. The percentage of positively stained cells in the articular cartilage and synovium was calculated (Additional file 1).

### Statistical analysis

Data were expressed as mean ± standard deviation. Differences between two groups were compared via two-sided Student’s t-test. Multifactorial comparisons were performed through one-way analysis of variance. Data with p < 0.05 were considered to have statistically significant differences. In this study, “*”and “**” denoted p < 0.05 and p < 0.01, respectively. The data analysis was calculated with SPSS 22.0 analysis software (SPSS Inc, Chicago, IL, USA).

### Supplementary Information

**Additional file 1: Figure S1.** miRNA expression profiling of macrophage-derived exosomes. **Figure S2.** miR-26b-5p overexpressed cell lines. Immunofluorescence and miRNA expression of lentivirus-transfected RAW264.7 cell line (**A**, **B**) and ATDC5 cell line (**C**, **D**). **Table S1.** Primer sequences used in the article. **Table S2.** Luminex liquid chip used for multi-cytokine detection.

## Data Availability

The data are available from the corresponding author on reasonable request.
