# Supplementary material for: M2 macrophage-derived exosomal miR-26b-5p regulates macrophage polarization and chondrocyte hypertrophy by targeting TLR3 and COL10A1 to alleviate osteoarthritis
Source: J Nanobiotechnology. 2024 Feb 19;22:72. doi: 10.1186/s12951-024-02336-4 (PMC10877765; doi:10.1186/s12951-024-02336-4)
Supplement: Supplementary file 1 — Additional file 1: Figure S1. miRNA expression profiling of macrophage-derived exosomes. Figure S2. miR-26b-5p overexpressed cell lines. Immunofluorescence and miRNA expression of lentivirus-transfected RAW264.7 cell line (A, B) and ATDC5 cell line (C, D). Table S1. Primer sequences used in the article. Table S2. Luminex liquid chip used for multi-cytokine detection. [file 12951_2024_2336_MOESM1_ESM.docx]

**Supplementary Information**

**M2 macrophage-derived exosomal miR-26b-5p regulates macrophage polarization and chondrocyte hypertrophy by targeting TLR3 and COL10A1 to alleviate osteoarthriti**

Yufan Qian^1†^, Genglei Chu^1,2†^, Lei Zhang^1†^, Zhikai Wu^1^, Qiuyuan Wang^1^, Jiong Jiong Guo^1*^, Feng Zhou^1,2*^

^1^ Department of Orthopaedics, The First Affiliated Hospital of Soochow University, Soochow University, Suzhou, Jiangsu, China.

^2^ Orthopedic Institute, Medical College, Soochow University, Suzhou, Jiangsu, China

^†^ These authors contributed equally to this work.

^*^ Corresponding authors:

Feng Zhou: Department of Orthopaedics, The First Affiliated Hospital of Soochow University. No. 899 Ping Hai Road, Suzhou, Jiangsu, China. E-mail: sdfyyzhoufeng@163.com.

Jiong Jiong Guo: Department of Orthopaedics, The First Affiliated Hospital of Soochow University. No. 899 Ping Hai Road, Suzhou, Jiangsu, China. E-mail: drjjguo@163.com.


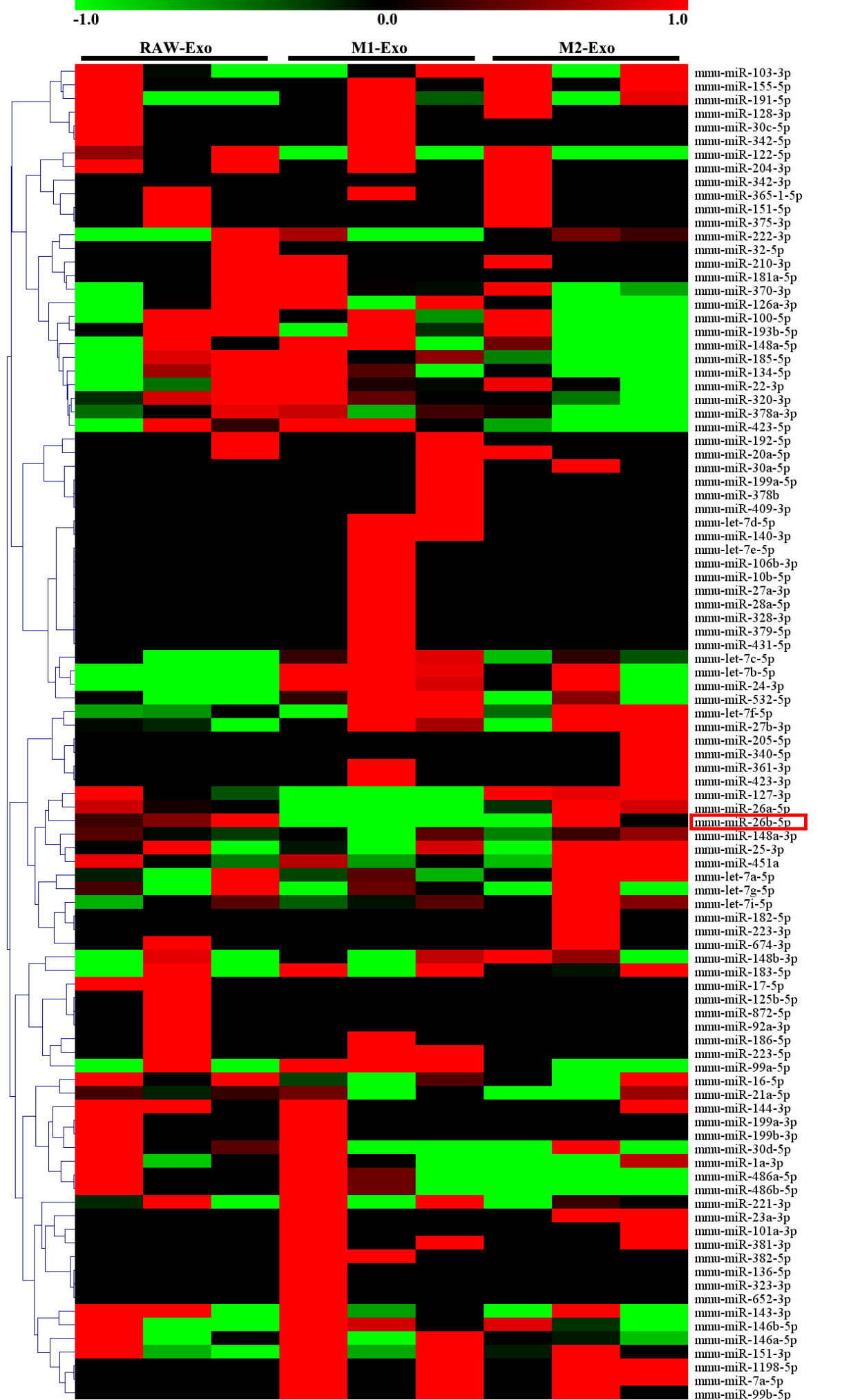


Figure S1. miRNA expression profiling of macrophage-derived exosomes.


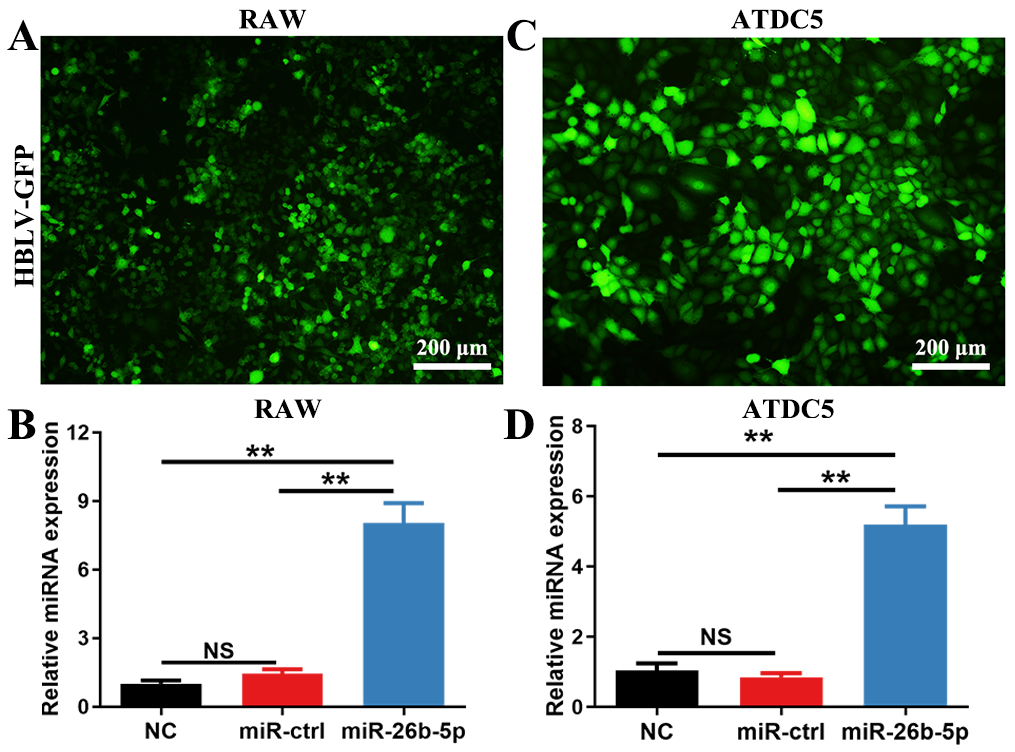


Figure S2. miR-26b-5p overexpressed cell lines. Immunofluorescence and miRNA expression of lentivirus-transfected RAW264.7 cell line (A, B) and ATDC5 cell line (C, D).

Table S1. Primer sequences used in the article**.**

| miR-134-5p RT Primer | | 5’-GTCGTATCCAGTGCGTGTCGTGGAGTCGGCAA  TTGCACTGGATACGACCCCCTC-3’ |
| --- | --- | --- |
| miR-134-5p-F | | 5’-GGGTGTGACTGGTTGACCA-3’ |
| miR-134-5p-R | | 5’-CAGTGCGTGTCGTGGAGT-3’ |
| miR-127-3p RT Prime | | 5’-GTCGTATCCAGTGCGTGTCGTGGAGTCGGCAA  TTGCACTGGATACGACAGCCAA-3’ |
| miR-127-3p-F | | 5’-GGGTCGGATCCGTCTGAGC-3’ |
| miR-127-3p-R | | 5’-CAGTGCGTGTCGTGGAGT-3’ |
| miR-26b-5p RT Primer | | 5’-GTCGTATCCAGTGCGTGTCGTGGAGTCGGCAA  TTGCACTGGATACGACACCTAT-3’ |
| miR-26b-5p-F | | 5’-GGGTTCAAGTAATTCAGG-3’ |
| miR-26b-5p-R | | 5’-CAGTGCGTGTCGTGGAGT-3’ |
| U6 RT Primer | 5’- AACGCTTCACGAATTTGCGT-3’ | |
| U6-F | 5’-CTCGCTTCGGCAGCACA-3’ | |
| U6-R | 5’-AACGCTTCACGAATTTGCGT-3’ | |
| IL-1β-F | 5’-TTCAAGGGGACATTAGGCAG-3’ | |
| IL-1β-R | 5’-TGTGCTGGTGCTTCATTCAT-3’ | |
| IL-6-F | 5ʹ-CAACGATGATGCACTTGCAGA-3ʹ | |
| IL-6-R | 5ʹ-TGTGACTCCAGCTTATCTCTTGG-3ʹ | |
| TNF-α-F | 5’-CTCAGCGAGGACAGCAAGG-3’ | |
| TNF-α-R | 5’-AGGGACAGAACCTGCCTGG-3’ | |
| Ptgs2-F | 5’-TTCAACACACTCTATCACTGGC-3’ | |
| Ptgs2-R | 5’-AGAAGCGTTTGCGGTACTCAT-3’ | |
| iNOS-F | 5’-GCGCTCTAGTGAAGCAAAGC-3’ | |
| iNOS-R | 5’-AGTGAAATCCGATGTGGCCT-3’ | |
| Arg-1-F | 5’-AGGCGCTGTCATCGATTTCT-3’ | |
| Arg-1-R | 5’-TGGAGTCCAGCAGACTCAAT-3’ | |
| RUNX-2-F | 5’-TCCCCGGGA ACCAAGAAGGCA-3’ | |
| RUNX-2-R | 5’-AGGGAGGGCCGTGGGTTCTG-3’ | |
| Col10a1-F | 5’-AAAGCTTACCCAGCAGTAGG-3’ | |
| Col10a1-R | 5’-ACGTACTCAGAGGAGTAGAG -3’ | |
| Col1a1-F | 5’-GCTCCTCTTAGGGGCCACT-3’ | |
| Col1a1-R | 5’-CCACGTCTCACCATTGGGG-3’ | |
| Col2a1-F | 5’-CACCCTCAA ATCCCTCAA CAATCA G-3’ | |
| Col2a1 -R | 5’-TGTCTTTCGTCTTGCTGGTCCACC-3’ | |
| SOX9-F | 5’-TACCTACGGCATCAGCAGCTC-3’ | |
| SOX9-R | 5’-TTGCCTTCACGTGGCTTTAAG-3’ | |
| Aggrecan-F | 5ʹ-CAGTGCGATGCAGGCTGGCT-3ʹ | |
| Aggrecan-R | 5ʹ-CCTCCGGCACTCGTTGGCTG-3ʹ | |

Table S2. Luminex liquid chip used for multi-cytokine detection.

|  | RWA264.7-CM (pg/ml) | M1-CM (pg/ml) | p value |
| --- | --- | --- | --- |
| Mo IL-1a | 3.94±0.58 | 25.94±4.01 | 0.001 |
| Mo IL-1b | 7.08±1.04 | 38.19±5.89 | 0.001 |
| Mo IL-2 | 3.93±0.57 | 13.13±2.02 | 0.002 |
| Mo IL-3 | 0.55±0.08 | 3.20±0.49 | 0.001 |
| Mo IL-4 | 1.6±0.23 | 4.705±0.73 | 0.002 |
| Mo IL-5 | 1.87±0.27 | 7.23±1.12 | 0.001 |
| Mo IL-6 | 1.31±0.19 | 1966.91±303.59 | 0.000 |
| Mo IL-9 | 5.88±0.86 | 23.65±3.65 | 0.001 |
| Mo IL-10 | 11.58±1.70 | 2069.40±319.42 | 0.000 |
| Mo IL-12(p40) | 1.72±0.25 | 52.91±8.17 | 0.000 |
| Mo IL-12(p70) | 28.47±4.17 | 116.36±17.96 | 0.001 |
| Mo IL-13 | 22.26±3.26 | 76.26±11.77 | 0.002 |
| Mo IL-17A | 2.01±0.29 | 7.49±1.16 | 0.001 |
| Mo Eotaxin | 4.14±0.61 | 32.10±4.95 | 0.001 |
| Mo G-CSF | 70.42±10.31 | 41309.00±6376.16 | 0.000 |
| Mo GM-CSF | 20.64±3.02 | 57.18±8.83 | 0.002 |
| Mo IFN-g | 2.32±0.34 | 252.60±38.99 | 0.000 |
| Mo KC | 4.19±0.61 | 14.67±2.26 | 0.002 |
| Mo MCP-1 | 679.75±99.54 | 12724.72±1964.09 | 0.000 |
| Mo MIP-1a | OOR > | OOR > | - |
| Mo MIP-1b | 22742.12±3330.36 | 31462.30±4856.29 | 0.062 |
| Mo RANTES | 181.78±26.62 | 7064.37±1090.41 | 0.000 |
| Mo TNF-a | 759.96±111.29 | 159787.88±24663.69 | 0.000 |
